# Histone post-translational modifications in frontal cortex from human donors with Alzheimer’s disease

**DOI:** 10.1186/s12014-015-9098-1

**Published:** 2015-10-01

**Authors:** Kyle W. Anderson, Illarion V. Turko

**Affiliations:** Institute for Bioscience and Biotechnology Research, Rockville, MD 20850 USA; Biomolecular Measurement Division, National Institute of Standards and Technology, Gaithersburg, MD 20899 USA; Department of Chemistry and Biochemistry, University of Maryland, College Park, MD 20742 USA

**Keywords:** Alzheimer’s disease, Histone, Multiple reaction monitoring, Frontal cortex

## Abstract

**Background:**

Alzheimer’s disease (AD) is the sixth leading cause of death and the most costly disease in the US. Despite the enormous impact of AD, there are no treatments that delay onset or stop disease progression currently on the market. This is partly due to the complexity of the disease and the largely unknown pathogenesis of sporadic AD, which accounts for the vast majority of cases. Epigenetics has been implicated as a critical component to AD pathology and a potential “hot spot” for treatments. Histone post-translational modifications (PTMs) are a key element in epigenetic regulation of gene expression and are known to be associated with the pathology of numerous diseases. Investigation of histone PTMs can help elucidate AD pathology and identify targets for therapies.

**Results:**

A multiple reaction monitoring mass spectrometry assay was used to measure changes in abundance of several histone PTMs in frontal cortex from human donors affected with AD (n = 6) and age-matched, normal donors (n = 6). Of the changes observed, notable decreases in methylation of H2B residue K108 by 25 % and H4 residue R55 by 35 % were measured and are likely associated with hydrogen bonding networks important for nucleosome stability. Additionally, a 91 % increase in ubiquitination of K120 on H2B was measured as well as an apparent loss in acetylation of the region near the N-terminus of H4. Our method of quantification was also determined to be precise and robust, signifying measured changes were representative of true biological differences between donors and sample groups.

**Conclusion:**

We are the first to report changes in methylation of H2B K108, methylation of H4 R55, and ubiquitination of H2B K120 in frontal cortex from human donors with AD. These notable PTM changes may be of great importance in elucidating the epigenetic mechanism of AD as it relates to disease pathology. Beyond the structural and functional impacts of the changes we have measured, the sites of altered PTMs may be used to identify enzymes responsible for their modulation, which could be used as prospective drug targets for highly specific AD therapies.

**Electronic supplementary material:**

The online version of this article (doi:10.1186/s12014-015-9098-1) contains supplementary material, which is available to authorized users.

## Background

DNA is packaged into the eukaryotic nucleus as chromatin, a structure comprised of nucleosomes formed by 146 base pairs of DNA wrapped around an octamer of duplicates of histones H2A, H2B, H3, and H4. These histones are dynamically modified by post-translational modifications (PTMs), known as the “histone code”, which can regulate gene expression. Disruption of these PTM codes through the use of inhibitors can affect cell cycle, alter gene expression, and induce apoptosis [1]. Alzheimer’s disease (AD) is the leading neurodegenerative disorder and characterized by a global repression of gene expression. Studies on monozygous twins suggest that AD may be caused by epigenetic factors, an explanation for different outcomes when the DNA sequence is conserved [2]. Once acquired, epigenetic factors, such as DNA methylation and histone PTMs, can be passed on to offspring cells [3]. Common PTMs in histones are acetylation, methylation, phosphorylation, and ubiquitination. Histone PTMs are classified into two main mechanisms of function: *cis* and *trans*. *Cis* mechanisms modify interactions within and between nucleosomes, while *trans* mechanisms act as signals for other proteins to assist in alteration of gene expression, chromatin, or other cellular functions [3]. Acetylation of histones is generally characterized by an elevation in gene expression, conversely, deacetylation is associated with a decrease in gene expression, which is a commonality in AD [4]. Methylation of arginine and lysine is involved in transcription regulation, chromatin remodeling, DNA repair, and signal transduction [5, 6]. Unlike histone acetyltransferases, histone methyltransferases are considerably more site specific and often are responsible for modifying only a single site on histones. Thus it is important to identify the specific sites of histone methylation and their changes relevant to pathology. Ubiquitination of histone residues has the potential to alter the affinity of histone–histone interactions within the nucleosome [7], indicate the presence of DNA damage [8, 9], and be a marker for other functional roles. Phosphorylation can be used to regulate mitosis and structural dynamics of nucleosomes. However, phosphorylation is labile and typically lost during the relatively long post-mortem interval in human tissues and therefore it is more appropriate to measure phosphorylation in animal models when the post-mortem interval is a few minutes [10].

In this study, we have identified several sites of histone PTMs in frontal cortex from human donors with AD that are differentially abundant compared to age-matched normal donors. The majority of PTM and protein measurements in AD research are performed in animal models of neurodegeneration [10, 11], with few studies being performed using human brain. While we do not underestimate the potential of animal models, measurements need to be performed in human brain tissue affected by AD to confirm changes are truly indicative of AD pathology. With our multiple reaction monitoring (MRM) mass spectrometry method of PTM quantification, we were able to measure several histone PTMs in frontal cortex from humans. Frontal cortex is an ideal neural tissue for investigation of PTMs because it is responsible for short-term memory, cognition, and decision making, which are all affected by AD, and it is severely damaged in late AD [12]. Significant changes in methylation of H2B K108 and H4 R55 and ubiquitination of H2B K120 reported herein have not been previously reported in frontal cortex from human donors affected with AD. Structural and functional effects from these modifications likely have implications in AD pathology and may identify enzymes associated with these PTMs to be used as drug targets.

## Methods

The DC Protein Assay kit was purchased from Bio-Rad Laboratories (Hercules, CA, USA). RapiGest SF surfactant was from Waters (Milford, MA, USA), Trypsin (T0303, Type IX-S from porcine pancreas) and all other chemicals were purchased from Sigma-Aldrich (St. Louis, MO, USA).

### Human tissue

Authors received frozen samples of frontal cortex from Washington University School of Medicine Alzheimer’s Disease Research Center (St. Louis, MO, USA). Human frontal cortex was collected in accordance with guidance from the Washington University Human Research Protection Office (HRPO number: 89-0556). The authors consulted the Washington University HRPO, which determined that Institutional Review Board (IRB) oversight was not required for this study and waived the need for IRB approval. In the state of Missouri, individuals can give prospective consent for autopsy; our participants provided this consent by signing the hospital’s autopsy form. If the participant does not provide future consent before death, the DPOA or next of kin provide it after death. Handling of tissues for sample processing conformed to University of Maryland regulations. All data were analyzed anonymously. Demographic information on the de-identified donors is summarized in Additional file 1: Table S1.

### Purification of histones from frontal cortex for PTM screening

To reduce sample complexity for data-dependent screening for PTMs, histones were purified from tissue. Frontal cortex tissue from both AD-affected and normal donors were carefully cleaned from white matter and blood and 1.1 g of cortex was minced. Nuclei isolation and histone purification was performed as previously described [13] using nuclei isolation buffer (NIB-250) composed of 15 mmol/L Tris–HCl (pH 7.5), 15 mmol/L NaCl, 60 mmol/L KCl, 5 mmol/L MgCl_2_, 1 mmol/L CaCl_2_, and 250 mmol/L sucrose. Tissue was added to 10 mL of NIB-250 containing 10 mmol/L DTT, 10 mmol/L sodium butyrate, and 0.2 % NP-40 and homogenized on ice with a glass/Teflon dounce homogenizer using 10 strokes. Homogenate was incubated for 10 min on ice and then centrifuged at 1000*g* for 7 min at 4 °C. Pellet was washed with NIB containing 10 mmol/L DTT and 10 mmol/L sodium butyrate, but no NP-40. Pellet was supplemented with 8 mL 0.4 mol/L H_2_SO_4_ and incubated for 2 h at 4 °C with rotation. Sample was then centrifuged at 3400*g* for 5 min at 4 °C and resulting supernatant was supplemented in a 4:1 (volume ratio) with trichloroacetic acid and allowed to precipitate overnight at 4 °C. Pellet was washed with 12 mL of acetone acidified with 0.1 % volume of hydrochloric acid and centrifuged for 5 min at 3400*g* at 4 °C. Pellet was washed with 12 mL of pure acetone and centrifuged for 5 min at 3400*g* at 4 °C. Pellet was air dried for 2 h at room temperature. 1 mL of water was added to the pellet and sample was gently rotated to allow for histones to be solubilized in the water. Sample was then centrifuged for 5 min at 3400*g* at room temperature and histones were collected in the supernatant. Histone concentration was determined using the DC protein assay kit and bovine serum albumin as a standard. Histones were stored at −20 °C before further processing. The resulting 100 µL histone sample (15.5 µg) was supplemented with 100 µL of 25 mmol/L NH_4_HCO_3_ and 0.1 % (mass fraction) RapiGest SF (Waters, Milford, MA, USA) then treated in a 25:1 mass ratio of histone to trypsin overnight at 37 °C. Following trypsinolysis, 0.5 % trifluoroacetic acid (TFA) was added and incubated at 37 °C for 1 h to cleave acid-labile RapiGest. Samples were then centrifuged at 179,000*g* for 30 min at 4 °C to remove precipitated surfactant. Supernatants were dried using a Vacufuge (Eppendorf AG, Hamburg, Germany).

### PTM identification and selection

Data-dependent acquisition of histone peptides was performed by LC–MS/MS using an Agilent 6550 QTOF. Peptides were eluted from an Agilent ProtID C18 nanochip (75 µm × 150 mm, 300 nm) over 120 min gradient from 3 to 35 % acetonitrile containing 0.1 % (volume fraction) formic acid at a flow rate of 300 nL/min. Acquisition method in positive mode used capillary temperature 275 °C, fragmentor 180 V, capillary voltage 1950 V, a 300–2000 *m*/*z* mass window at 8 spectra/s scan rate for precursor ions, 1.3 *m*/*z* isolation window, and a 80–1700 *m*/*z* mass window at 3 spectra/s scan rate for product ions. Collision energy was determined using the formula, CE = (3 *m*/*z*)/100 + 1, for 2+ charged peptides and the formula, CE = (3.6 *m/z*)/100 − 4.8, for peptides 3+ charged or greater.

Data was searched against the SwissProt database for human proteins using both Mascot (MatrixScience) and MassMatrix [14] to identify histone peptides and PTMs. Search settings were: precursor mass tolerance, 0.05 Da; fragment mass tolerance, 0.05 Da; maximum missed cleavages, 4; variable modifications, acetylation of lysine, methylation of arginine and lysine, and ubiquitination of lysine; enzyme, trypsin. Both Mascot and MassMatrix were used in parallel to reduce false positives based on inherent differences of individual searching algorithms by comparing identifications and their respective retention times in both software platforms for a consensus. Identifications in consensus with both Mascot and MassMatrix were manually confirmed by spectra annotation. MassMatrix also provides LC retention time analysis, which calculates the theoretical retention time and compares it the observed retention time and displays a confidence score. Retention time confidence scores less than 1 % were removed, as recommended by MassMatrix [15]. Fragmentation data was then used to develop a transition list for MRM (Additional file 2: Table S2).

### Human frontal cortex processing for quantitative measurements

For quantitative measurements, samples were minimally processed (1) to reduce loss of native PTMs and (2) to reduce PTM artifacts caused by sample processing. Frontal cortex tissue was carefully cleaned from white matter and blood from AD-affected (n = 6) and normal (n = 6) donors. Tissue (0.2 g) was minced and then homogenized in 1 mL of 25 mmol/L NH_4_HCO_3_ by sonication at 50 W using four 10 s continuous cycles (Sonicator 3000, Misonix Inc., Farmingdale, NY, USA). Homogenates were centrifuged at 2000*g* for 5 min to remove debris. Resulting supernatant was measured for total protein concentration using detergent-compatible DC protein assay kit in the presence of 1 % (mass fraction) SDS and bovine serum albumin as a standard. Homogenates were stored at −80 °C. Samples of 0.2 mg were supplemented with 20 mmol/L DTT and 1 % (mass fraction) SDS. After 1 h incubation at room temperature to reduce cysteines, 55 mmol/L iodoacetamide was added and incubated for an additional 1 h to alkylate cysteines. Protein was isolated by chloroform/methanol precipitation. Protein pellets were sonicated in 1 mL 25 mmol/L NH_4_HCO_3_ and 0.1 % (mass fraction) RapiGest SF and then treated with a 25:1 mass ratio of histone to trypsin overnight at 37 °C. Following trypsinolysis, 0.5 % TFA was added and incubated at 37 °C for 1 h to cleave acid-labile RapiGest. Samples were then centrifuged at 179,000*g* for 30 min at 4 °C to remove precipitated surfactant. Supernatants were dried using a Vacufuge.

### PTM quantification by multiple reaction monitoring

Dried peptides were reconstituted in 3 % acetonitrile, 97 % water, and 0.1 % formic acid (volume fraction). Separation was performed on an Agilent Zorbax Eclipse Plus C18 RRHD column (2.1 mm × 50 mm, 1.8 µm particle) and multiple reaction monitoring (MRM) analysis was performed on an Agilent 6490 iFunnel Triple Quadrupole LC/MS system (Santa Clara, CA, USA). Peptides were eluted at a flow rate of 200 µL/min using the following gradient of solvent B in solvent A: 3 % B for 3 min, 3–30 % B in 32 min, 30–80 % B in 5 min, and 80–3 % B in 3 min. Solvent A was water containing 0.1 % (volume fraction) formic acid and solvent B was acetonitrile containing 0.1 % formic acid. The acquisition method used the following parameters in positive mode: fragmentor 380 V, collision energy 25 V, cell accelerator 4 V, electron multiplier 600 V, and capillary voltage 3500 V. Selected transitions from fragmentation data and nonlinear retention time alignment from data-dependent MS analysis were used to confirm the retention time and identity during MRM analysis (Additional file 2: Table S2); the transition most appropriate for quantification for each peptide is listed in Table 1. MRM assay development met applicable criteria for Tier 3 MRM assay [16].

**Table 1:** Histone modifications and associated MRM transitions used for quantification

Histone	Sequence	Precursor ion (*m*/*z*)	Product ion (*m*/*z*)
H2A	*AGLQFPVGR*	472.796 (2+)	703.39 (1+, y6)
H2A (canonical)	VTIAQGGVLPNIQAVLLPK	966.08 (2+)	1092.68 (1+, y10)
H2A (1-A, 2-B, H2Ax)	LLGGVTIAQGGVLPNIQAVLLPK	757.80 (3+)	546.84 (2+, y10)
*H2B*	*EIQTAVR*	408.73 (2+)	446.27 (1+, y4)
H2B, K108-methylation	LLLPGELAKme	484.31 (2+)	736.46 (1+, b7)
H2B, K120-ubiquitination	AVTKubYTSSK	549.79 (2+)	827.43 (1+, y6)
*H3*	*DIQLAR*	358.21 (2+)	359.24 (1+, y3)
H3, K4-, K9-acetylation	KacQLATKacAAR	535.82 (2+)	772.47 (1+, y7)
*H4*	*VFLENVIR*	495.29 (2+)	501.31 (1+, y4)
H4, K8-, K12-, K16-acetylation	GGKacGLGKacGGAKacR	606.34 (2+)	927.54 (1+, y9)
H4, K12-, K16-acetylation	GLGKacGGAKacR	464.27 (2+)	530.30 (1+, y5)
H4, R55-methylation	ISGLIYEETRme	597.82 (2+)	290.18 (1+, y2)

Modifications (ac) acetylation, (me) methylation, and (ub) ubiquitination of peptides were measured and normalized to an unmodified peptide, shown in italics, from the respective histone

Chromatographic peaks were integrated for transitions selected for quantification and peak areas for peptides were divided by the peak area of a non-modified peptide for the respective histone (Table 1). Ratios of biological replicates and injection replicates were averaged and the average from the normal frontal cortex samples was used to normalize the normal group to 1.00 and to normalize the AD-affected group for easy comparison of fold changes. Student’s two-tailed *t* test was performed to determine the level of statistical significance of changes between AD and normal groups.

## Results and discussion

### Selection of transitions for MRM

Initially, histones were purified from normal and AD-affected tissue to produce a sample with less complex matrix for improved identification of histone peptides in a discovery mode screening. While dynamic exclusion was used during MS acquisition, data-dependent analysis benefits from having enrichment of the histone targets, particularly when a histone PTM may be only a few percent of the total amount of the unmodified peptide. Once peptides were identified by Mascot and MassMatrix and manually confirmed using retention time analysis and spectra annotation, a transition list was created from abundant fragment ions, which was used for MRM. The optimized transitions used for quantification are in Table 1.

### Preparation of samples

SDS was added to tissue homogenates at the very first step of sample processing to arrest enzymatic activity and minimize unwanted loss of PTMs. Early addition of SDS also efficiently denatures proteins and evenly exposes Cys residues in all samples for subsequent reduction and alkylation. A challenge to processing brain tissue for MS analysis is its high lipid content, which can degrade chromatography performance and contribute to ion suppression. The whole brain is approximately 80 % lipid by dry mass [17] and, therefore, necessitates removal of lipids. Chloroform/methanol precipitation of the protein efficiently removed lipids, SDS, salts, and by-products alkylation to yield a pure protein pellet. For PTM screening, histones were extensively purified. However, it was preferable to minimally process the tissue samples for quantification to evaluate differences in AD versus normal frontal cortex because extensive processing may cause a loss in PTMs and also introduce processing artifacts, resulting in inaccuracy of measurements [18, 19]. These two major steps, (1) early addition of SDS followed by reduction/alkylation of Cys residues and (2) chloroform/methanol precipitation to increase protein purity, ensure sample quality compatible with LC–MS/MS analyses, while minimizing processing to maintain endogenous PTMs.

### Reproducibility and data quality

**Fig. 1:**
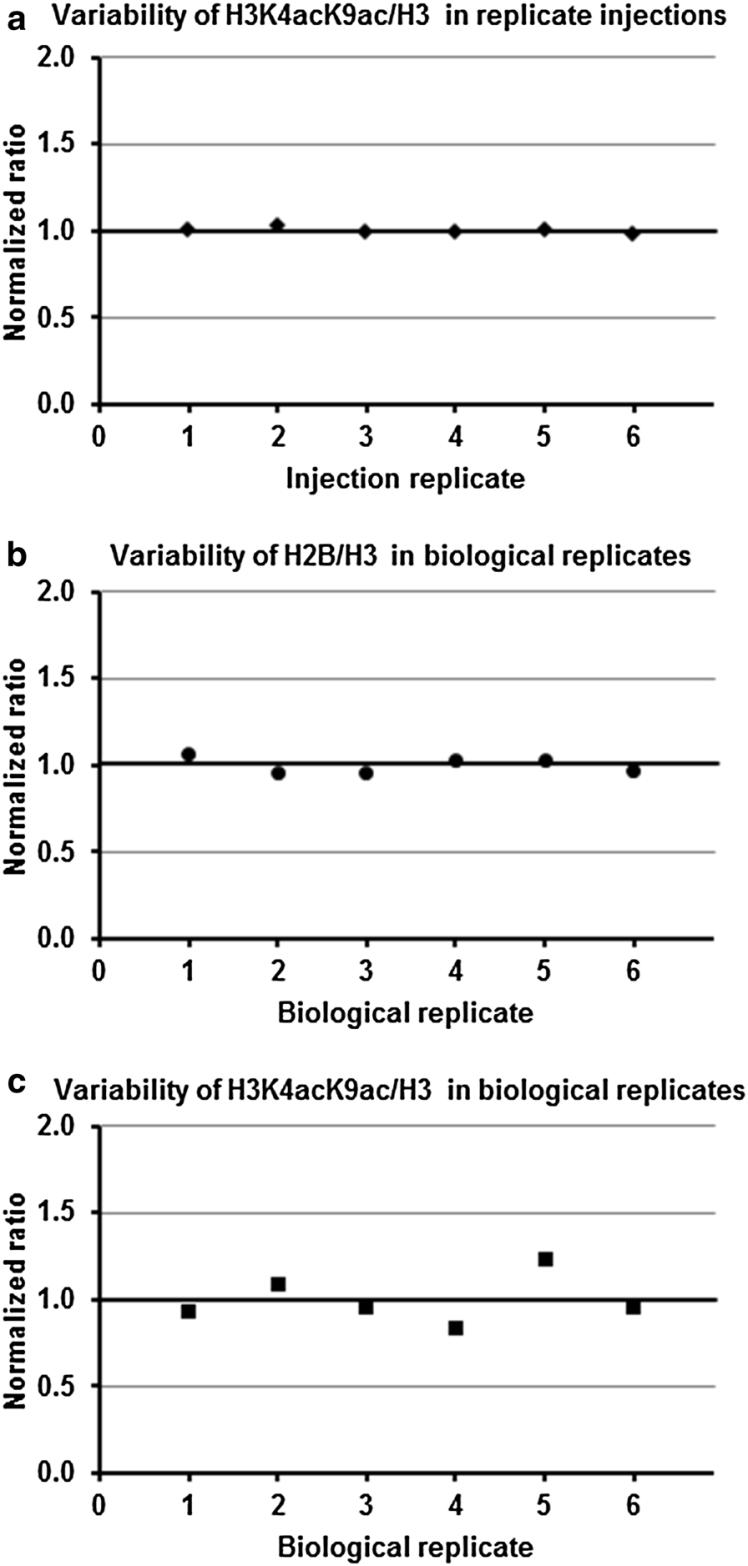
Variability in replicate measurements. **a** The ratio of modified H3 peptide KacQLATKacAAR to H3 was measured in six replicate injections of a frontal cortex sample from a normal donor and individual ratios were normalized to their average (n = 6) and plotted to show the negligible variability between replicate injections. **b** The ratio of non-modified H2B to H3 plotted for biological replicates from normal donors (n = 6) and normalized to the average, showing negligible variability between biological replicates for the stoichiometric H2B/H3 ratio. **c** The ratio of KacQLATKacAAR to H3 plotted for the average of two injections for each biological sample from normal donors (n = 6) and normalized to the average for all measurements (n = 12), showing variability in PTM is attributed to differences in donors and not due to inherent variability in MRM

Selected transitions for quantification had Gaussian peak shape with more than ten data points across each peak and suitable intensity as is preferred for quantification (Additional file 3: Figure S1). However, it is important to establish the level of variability in measurements to confirm that observed changes are attributed to true biological changes and not an artifact of LC–MS/MS performance or sample preparation. To demonstrate our data quality, we first performed six replicate injections of the same sample (Fig. 1a) and measured modified H3 peptide KacQLATKacAAR as a ratio to non-modified H3 peptide DIQLAR. Measured ratios were nearly identical with negligible variation (Fig. 1a), demonstrating LC–MS/MS performance was precise and not a contributor to measurement variation. Second, we compared variability between six biological samples from normal frontal cortex using the ratio of non-modified H2B peptide EIQTAVR to H3 peptide DIQLAR (Fig. 1b). Histones H2B and H3 are present in equal amounts as there is a 1:1 stoichiometry between core histones in the nucleosome. Therefore, H2B to H3 should not change between donors or sample preparations. Indeed, our measurements confirm that there is negligible variation in H2B/H3 between donors, demonstrating that our sample preparation results in precision of measurements. Lastly, we monitored the H3 peptide KacQLATKacAAR as a ratio to H3 peptide DIQLAR (Fig. 1c) for the same six donors of normal frontal cortex and in the same LC–MS/MS runs as for Fig. 1b. Detectable variability across donors for KacQLATKacAAR shows that variability in KacQLATKacAAR measurements is due to biological differences in PTM level between donors. In summary, we established that measured differences between AD and control samples described herein are representative of biological variation and that our sample preparation and MRM assay is robust and precise.

### Effect of histone used for normalization

**Fig. 2:**
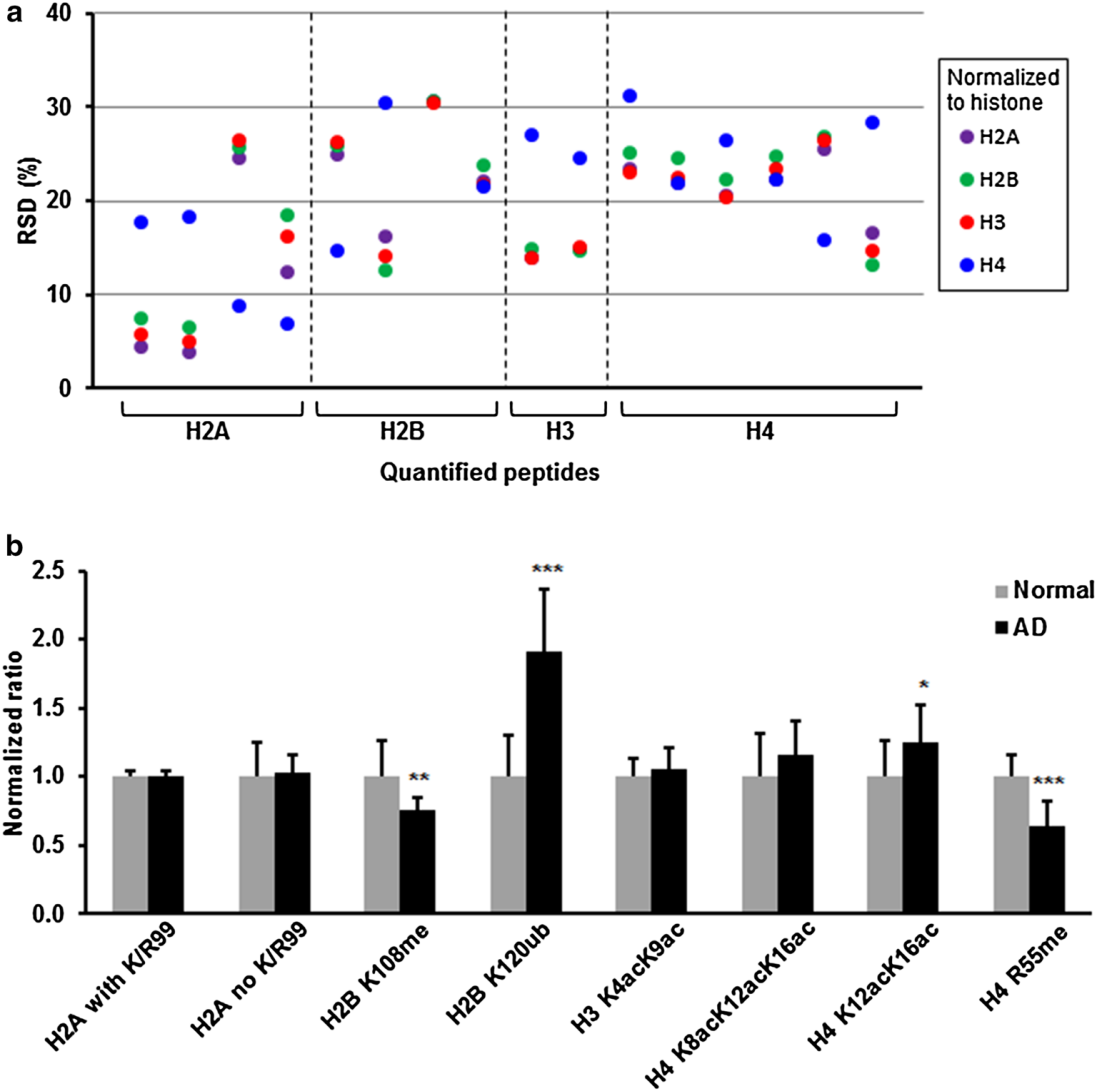
Histone PTMs in frontal cortex. **a** Measured peak areas of each quantified peptide were normalized to the peak area of different histone subunits. The relative standard deviation (n = 12) for each quantified peptide in normal and AD frontal cortex is shown grouped by the histone in which the peptides are located. The identity of the PTM measurements is presented in the *graph* in **b**, which appears in the same order. **b** Changes in histone PTMs in AD-affected human frontal cortex. Measurements were performed using human frontal cortex from normal (n = 6) and AD-affected (n = 6) donors (Additional file 1: Table S1) and two experimental replicates per donor (n = 12 total measurements per condition). Q-peptide transitions are summarized in Table 1. Data presented as mean ± SD. PTMs are (ac) acetylation, (me) methylation, and (ub) ubquitination. *p < 0.05; **p < 0.01; ***p < 0.001

Changes in quantified histone PTMs between AD and normal frontal cortex from human donors are presented in Fig. 2b. For quantification of histone PTMs, the peak areas of modified peptides were normalized to the peak areas of non-modified peptides from the respective histone. For example, ubiquitinated H2B peptide AVTKubYTSSK was normalized to H2B peptide EIQTAVR while acetylated H4 peptide GLGKacGGAKacR was normalized to H4 peptide VFLENVIR. While normalization of PTMs within a protein to the total amount of the protein is an accepted practice for quantification [20], it is typically not advisable to normalize PTMs within one protein to the total abundance of a different protein. Histones, however, are present in a stoichiometric 1:1:1:1 ratio of H2A:H2B:H3:H4 in the nucleosome. Due to their equal abundance, using a peptide from a single histone for normalization of all measured histone PTMs should generate similar quantification results and relative standard deviation (RSD) without a bias for using the respective histone that contains the PTM for normalization. We performed a series of normalizations, normalizing all measured PTMs to a single histone to evaluate the effect of normalization (Fig. 2a). We were able to show that there is a close consensus in RSD between H2A, H2B, and H3 normalization. H4 normalization varied in RSD with H4 RSD matching that of the other histones or being more or less than that of the other histones used for normalization. While H4 was often different than the consensus RSD for other histones, it did not show bias for H4 PTMs or PTMs of other histones. While the histone used for normalization should not have an effect, we would expect any effect on normalization to have a bias for normalizing PTMs to their respective histone, resulting in lower RSD for H4 PTMs to total H4 pairs. Additionally, no single histone showed a significantly different RSD when used for normalization. The average RSD when PTMs were normalized to their respective histones was 19 %, while normalizing all PTMs to one histone yielded an average RSD of 19 % for H2A, 20 % for H2B, 19 % for H3, and 22 % for H4. Overall, we were not able to observe any bias in histone normalization, which supports the stoichiometric relationship of histones in the nucleosome and the quality of our normalized measurements.

### Implications of histone PTMs in AD-affected human frontal cortex

Observed changes in histone PTMs in AD are presented in Fig. 2b. In most sequence variants, histone H2A contains a lysine or arginine residue at position 99, however variants 1-A, 2-B, and H2Ax do not contain K/R99. Since K/R99 is a potential site for modification, the inability to regulate by K/R99 via a reduction in variants that contain this site could be of importance. We did not observe any differences in abundance in isoforms with K/R99 or without K/R99, indicating turnover of these variants is not present in AD pathology in the frontal cortex. Similarly, measurements for K4- and K9-acetylated H3 and K8-, K12-, and K16-acetylated H4 did not show statistically significant changes between AD and control. We did measure significant changes in PTMs of other sites that are associated with structural and functional implications related to AD.

K12- and K16-acetylated H4 increased 25 % in AD. The increase in K12- and K16-acetylated H4 could be due to a loss in acetylation of K8, leading to tryptic cleavage after K8 and increasing the peptide ^9^GLGKacGGAKacR^17^. While we did not observe a change in ^6^GGKacGLGKacGGAKacR^17^, loss of K5 acetylation may have increased fragment 6–17, which was simultaneously undergoing a loss of K8 acetylation, resulting in no net change in fragment 6–17. Global deaceylation of histones is associated with AD; however, our data suggests that deacetylation may be occurring more frequently at the N-terminus of the histone tail. The N-terminal regions of histones are rich in glycine residues, which provide a high degree of flexibility to histone tails and lack of tertiary structure. Additionally, the high abundance of basic residues lysine and arginine contribute to an overall positive charge. Positively charged histone tails and negatively charged DNA form an electrostatic interaction that structurally results in the tail region wrapping along the DNA backbone, thereby forming a tight nucleosome structure [21]. The reduction in net charge, due to the DNA-histone tail interaction, and orientation of the extended tail against the nucleosome surface lead to denser, less transcriptionally active chromatin [21]. The loss of N-terminal acetylation induces this charge interaction and likely results in decreased gene expression in the AD frontal cortex. Deacetylation of histones is catalyzed by histone deacetylases (HDACs) and there is a growing interest in using HDAC inhibitors to slow aberrant deacetylation in brain [22]. Our previous work measured absolute concentrations of HDAC isoforms and identified specific isoforms associated with AD pathology in various neural tissues [4].

**Fig. 3:**
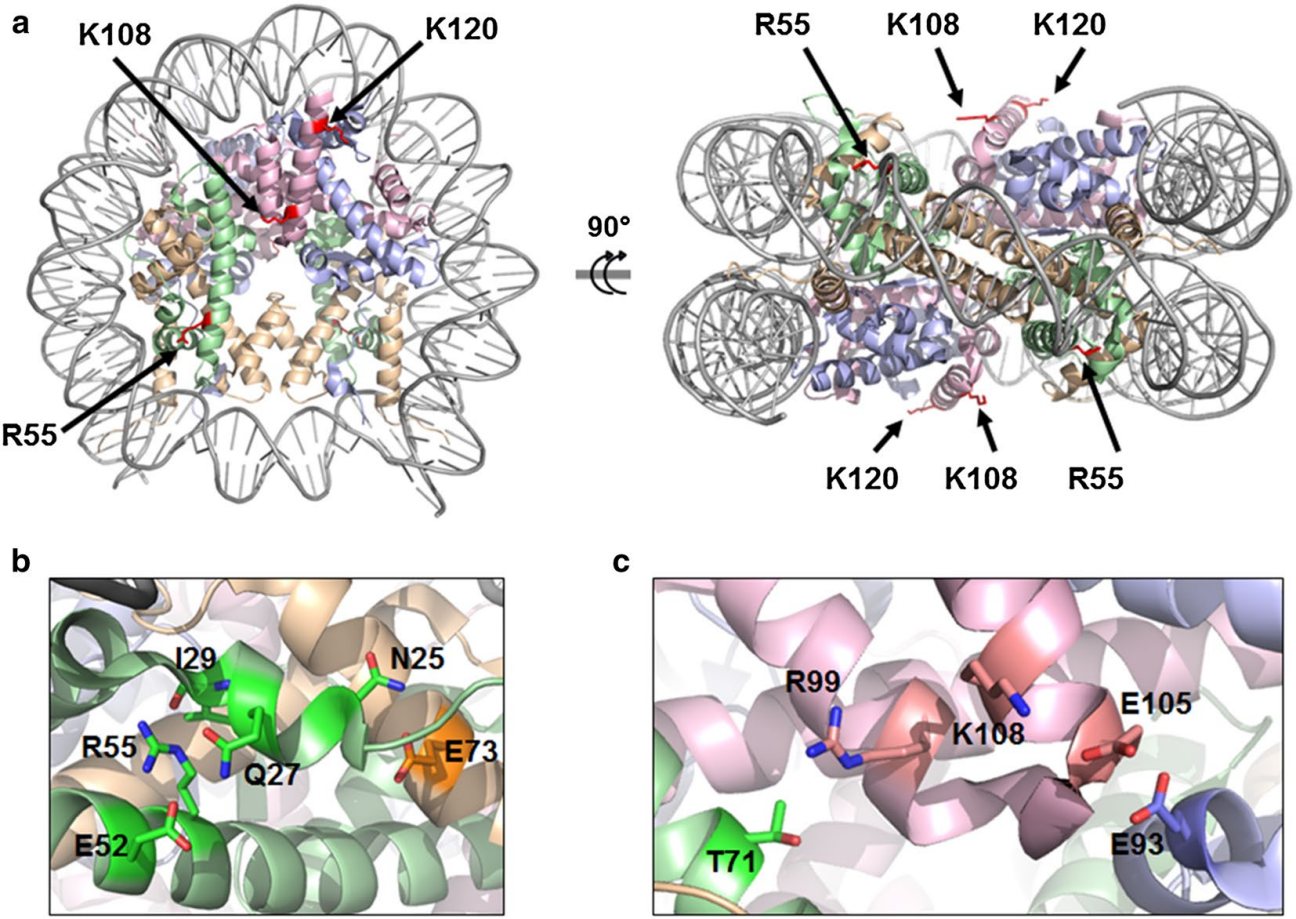
Location of core PTMs on nucleosome surface. H2A (*blue*), H2B (*pink*), H3 (*yellow*), and H4 (*green*) form an octamer in the nucleosome core, which is wrapped by DNA. Nucleosome crystal structure PDB ID: 2CV5 [25] was modified using PyMOL Molecular Graphics System (Version 1.7.2, Schrödinger, LLC). **a** Methylation sites R55 on H4 and K108 on H3 and ubiquitination site K121 on H2B are shown in *red*. **b** Hydrogen bond network of H4 R55. **c** Hydrogen bond network of H2B K108

While HDAC has demonstrated potential as a prospective AD therapy, therapies targeting other histone-modifying enzymes have yet to come forward. Other PTMs, such as methylation, are modulated by enzymes with greater residue specificity than acetyltransferases and deacetylases. Often times, a methyltransferase or demethylase may only act on one site in a histone [23]. This specificity may correlate to improved efficacy and safety when targeting enzymes for specific methylation sites compared to HDAC inhibition alone. Additionally, they may be more efficacious as a combinatorial treatment. H2B methylation at K108 decreased 25 % and H4 methylation at R55 decreased 35 % in AD frontal cortex (Fig. 2b). Both of these sites have been reported to be methylated in human cells [24]. To our knowledge, H2B K108 and H4 R55 methylation have not been investigated in AD-affected human frontal cortex. Both H2B K108 and H4 R55 are located on the outermost surface of the nucleosome (Fig. 3a) [25], suggesting their accessibility to methyltransferases and demethylases may incline them to be used as switches for transcriptional activity. Monomethylation is commonly a marker of transcription activation. Since AD is characterized by a global repression in gene expression, our observed decrease in several methylation sites strongly suggests their role in reducing transcription consistent with AD. H4 R55 participates in a complex hydrogen bond network in the nucleosome (Fig. 3b) [25]. H4 residues Q27 and E52 and the I29 backbone form a hydrogen bond network that structurally thrusts N25 into a hydrogen bond with E73 on neighboring histone H3 [26]. By forming a hydrogen bonding network that increases affinity between H3 and H4, the nucleosome becomes structurally more stable. However, methylation of H4 R55 likely disrupts this bonding network, thereby lessening the H3–H4 interaction and weakening the nucleosome [26]. Our observed decrease in methylation of R55 may be a mechanism for increasing nucleosome stability and favoring denser chromatin, which decreases gene expression. Similarly, H2B K108 is central to a hydrogen bond network involving histones H2A, H2B, and H4 (Fig. 3c) [25]. H2B K108 and E105 participate in intra-chain hydrogen bonding which structurally positions H2B residues for two interactions between neighboring histones favoring nucleosome stability. First, K108 forms a hydrogen bond with E105, pulling E105 away from close proximity to E93 on histone H2A. E105 and E93 can create electrostatic repulsion, however when E105 is distanced from E93 and hydrogen bonded to K108, this electrostatic repulsion is greatly reduced and H2A and H2B are able nestle together [25]. Second, the K108 hydrogen bond with E105 twists the helix-loop-helix, shown in Fig. 3c, to position R99 on H2B directly facing T71 on H4 for a hydrogen bond. This hydrogen bond between residues on H2B and H4 strengthen the H2B–H4 stability. Methylation likely disturbs this H4–H2B–H2A inter-chain network and would favor disassembly of the nucleosome. The structural and functional effect of our observed decrease in methylation of H2B K108 is consistent with the decrease in H4 R55, suggesting methylation of histones is used to down regulate transcriptional activity and gene expression in AD pathology. Establishing specific sites of histone methylation and their modulation, as described herein, is important in characterizing AD pathology.

H2B ubiquitination at K120 increased 91 % in AD (Fig. 2b). K120 has been reported to be ubiquitinated in human cell culture and animal neural tissue [11, 24, 27], but we were unable to find reports measuring ubiquitination of this site in AD-affected human frontal cortex. Polyubiquitination of proteins is commonly to mark proteins for degradation; however, monoubiquitination of proteins is typically used for regulatory functions [8]. After trypsin digestion, a di-glycine remnant of ubiquitination remains on modified lysine residues, which does not allow for differentiation between mono- and polyubiquitination. Polyubiquitination of histones is less common, but has been suggested to affect the affinity of histone–histone interactions within the nucleosome [7]. Monoubiquitination of histones has been shown to indicate the presence of DNA damage [8, 9] and to engage in “cross-talk” with methylation of other sites [27]. Ubiquitination of H2B K120 has been shown to be inversely related to methylation of H3 core residue K79 [27]. While we did not detect methylation of H3 K79, we did detect a decrease in methylation of other core residues H2B K108 and H4 R55. Additionally, site H2B K120 is an easily accessible site for ubiquitin ligases as it is located on the outermost surface of the nucleosome core and distant from the histone–DNA interface (Fig. 3a) [25], an important consideration for a relatively large, 8.5 kDa PTM. Abnormal accumulation of ubiquitinated proteins other than histones in AD has been reported [28], suggesting ubiquitination in AD may extend beyond histones and the epigenome.

## Conclusion

Histone PTMs have been implicated in many biological functions and diseases and serve an important role in epigenetic regulation of gene expression. Aberrant modulations in histone PTMs have been suggested to occur in brain as part of AD pathology. Histone PTMs that were found to be significantly different in AD frontal cortex were decreases in methylation of H2B K108 and H4 R55 and an increase in ubiquitination of H2B K120. Changes of these sites have not been previously reported in AD-affected frontal cortex in humans. Additionally, acetylated H4 peptide ^9^GLGKacGGAKacR^17^ increased in abundance, which may indicate N-terminal loss of acetylation of K5 and K8. Structural effects induced by changes in these PTMs likely alter gene expression in the brain. Future work to evaluate the combinatorial effect of these PTMs on gene expression and other specific functional roles may prove to be important in elucidating the pathology of AD. Moreover, the PTMs described herein could be used to identify specific histone-modifying enzymes to serve as drug targets for the treatment of AD, a disease which currently does not have any approved therapies to slow disease progression in humans. The etiology of AD is not known and the etiology may vary between individual cases of sporadic AD; however, epigenetic regulation is directly responsible for dysfunction in gene expression, which is consistently observed across AD individuals. Ideally, treatment at the earliest stage of disease pathology would be preferred, but the unknown and possibly diverse etiology strongly supports the pharmaceutical intervention of a conserved component of AD pathology, namely histone PTMs.

## Additional files

10.1186/s12014-015-9098-1 Donor information.
10.1186/s12014-015-9098-1 MRM transitions used for identification.
10.1186/s12014-015-9098-1 Chromatograms of transitions used for quantification. Transitions for H2A (purple), H2B (green), H3 (red), and H4 (blue) with non-modified peptides used for normalization in the first column.

## Abbreviations

MRM: multiple reaction monitoring
PTM: post-translational modification
