# Supplementary material for: Histone post-translational modifications in frontal cortex from human donors with Alzheimer’s disease
Source: Clin Proteomics. 2015 Oct 1;12:26. doi: 10.1186/s12014-015-9098-1 (PMC4591557; doi:10.1186/s12014-015-9098-1)
Supplement: Supplementary file 1 — 10.1186/s12014-015-9098-1 Donor information. [file 12014_2015_9098_MOESM1_ESM.docx]

**Table S1**. Donor information.

| **Donor ID** | **Age (years)** | **Gender** | **Disease status** | **PMI (h)** |
| --- | --- | --- | --- | --- |
| 1 | 90 | F | Normal | 9.5 |
| 2 | 87 | M | Normal | 6.3 |
| 3 | 80.7 | F | Normal | 5.5 |
| 4 | 86.9 | M | Normal | 11.6 |
| 5 | 87.2 | M | Normal | 12 |
| 6 | 92.1 | F | Normal | 6 |
| 11 | 79 | M | Severe AD | 6 |
| 12 | 73.6 | F | Severe AD | 4 |
| 13 | 81.2 | F | Severe AD | 4 |
| 14 | 91 | F | Severe AD | 4.5 |
| 15 | 72 | M | Severe AD | 1 |
| 16 | 84 | F | Severe AD | 4.5 |

Alzheimer’s disease (AD) in frontal cortex was evaluated by the Clinical Dementia Rating (CDR). The composite score of CDR ranges from 0 to 3 with 0 for no dementia representing normal brain and 3 for severe dementia representing severe AD-affected brain (http://www.biostat.wustl.edu/~adrc/cdrpgm/index.html). Post mortem interval (PMI) is defined as the time between death and when the collected tissue was frozen. Frontal cortex was obtained from Washington University School of Medicine Alzheimer’s Disease Research Center.
