# Supplementary material for: Histone post-translational modifications in frontal cortex from human donors with Alzheimer’s disease
Source: Clin Proteomics. 2015 Oct 1;12:26. doi: 10.1186/s12014-015-9098-1 (PMC4591557; doi:10.1186/s12014-015-9098-1)
Supplement: Supplementary file 2 — 10.1186/s12014-015-9098-1 MRM transitions used for identification. [file 12014_2015_9098_MOESM2_ESM.docx]

**Table S2.** MRM transitions used for identification.

| **Histone** | **Sequence** | **Precursor ion**  **(m/z)** | **Product ion**  **(m/z)** |
| --- | --- | --- | --- |
| H2A | AGLQFPVGR | 472.796 (2+) | 816.47 (1+, y7) |
|  |  | 472.796 (2+) | 703.39 (1+, y6) |
|  |  | 472.796 (2+) | 575.33 (1+, y5) |
|  |  | 472.796 (2+) | 428.26 (1+, y4) |
| H2A (canonical) | VTIAQGGVLPNIQAVLLPK | 966.08 (2+) | 1205.76 (1+, y11) |
|  |  | 966.08 (2+) | 1092.68 (1+, y10) |
|  |  | 966.08 (2+) | 839.50 (1+, b9) |
|  |  | 966.08 (2+) | 726.41 (1+, b8) |
| H2A (1-A, 2-B, H2Ax) | LLGGVTIAQGGVLPNIQAVLLPK | 757.80 (3+) | 546.84 (2+, y10) |
|  |  | 757.80 (3+) | 455.77 (2+, b10) |
|  |  | 757.80 (3+) | 1092.68 (1+, y10) |
|  |  | 757.80 (3+) | 768.50 (1+, y7) |
| H2B | EIQTAVR | 408.73 (2+) | 687.41 (1+, y6) |
|  |  | 408.73 (2+) | 574.33 (1+, y5) |
|  |  | 408.73 (2+) | 446.27 (1+, y4) |
|  |  | 408.73 (2+) | 354.22 (1+, y3) |
| H2B, K108-methylation | LLLPGELAKme | 484.31 (2+) | 736.46 (1+, b7) |
|  |  | 484.31 (2+) | 623.38 (1+, b6) |
|  |  | 484.31 (2+) | 531.31 (1+, y5) |
|  |  | 484.31 (2+) | 474.29 (1+, y4) |
|  |  | 484.31 (2+) | 345.25 (1+, y3) |
| H2B, K120-ubiquitination | AVTKubYTSSK | 549.79 (2+) | 827.43 (1+, y6) |
|  |  | 549.79 (2+) | 585.29 (1+, y5) |
|  |  | 549.79 (2+) | 422.22 (1+, y4) |
| H3 | DIQLAR | 358.21 (2+) | 359.24 (1+, y3) |
|  |  | 358.21 (2+) | 229.12 (1+, b2) |
| H3, K4-, K9-acetylation | KacQLATKacAAR | 535.82 (2+) | 772.47 (1+, y7) |
|  |  | 535.82 (2+) | 659.38 (1+, y6) |
| H4 | VFLENVIR | 495.29 (2+) | 890.51 (1+, y7) |
|  |  | 495.29 (2+) | 743.44 (1+, y6) |
|  |  | 495.29 (2+) | 630.36 (1+, y5) |
|  |  | 495.29 (2+) | 501.31 (1+, y4) |
| H4, K8-, K12-, K16-acetylation | GGKacGLGKacGGAKacR | 606.34 (2+) | 927.54 (1+, y9) |
|  |  | 606.34 (2+) | 870.52 (1+, y8) |
|  |  | 606.34 (2+) | 530.30 (1+, y5) |
| H4, K12-, K16-acetylation | GLGKacGGAKacR | 464.27 (2+) | 757.43 (1+, y7) |
|  |  | 464.27 (2+) | 700.41 (1+, y6) |
|  |  | 464.27 (2+) | 530.30 (1+, y5) |
|  |  | 464.27 (2+) | 473.28 (1+, y4) |
| H4, R55-methylation | ISGLIYEETRme | 597.82 (2+) | 711.33 (1+, y5) |
|  |  | 597.82 (2+) | 548.27 (1+, y4) |
|  |  | 597.82 (2+) | 419.22 (1+, y3) |
|  |  | 597.82 (2+) | 290.18 (1+, y2) |
|  |  | 597.82 (2+) | 905.46 (1+, b8) |

Several fragment ions obtained from data-dependent MS identification for peptides with (ac) acetylation, (me) methylation, and (ub) ubiquitination were monitored in the MRM assay to verify identity. Highlighted transitions were selected for quantification.
