# Supplementary figures and images for: Histone post-translational modifications in frontal cortex from human donors with Alzheimer’s disease
Source: Clin Proteomics. 2015 Oct 1;12:26. doi: 10.1186/s12014-015-9098-1 (PMC4591557; doi:10.1186/s12014-015-9098-1)

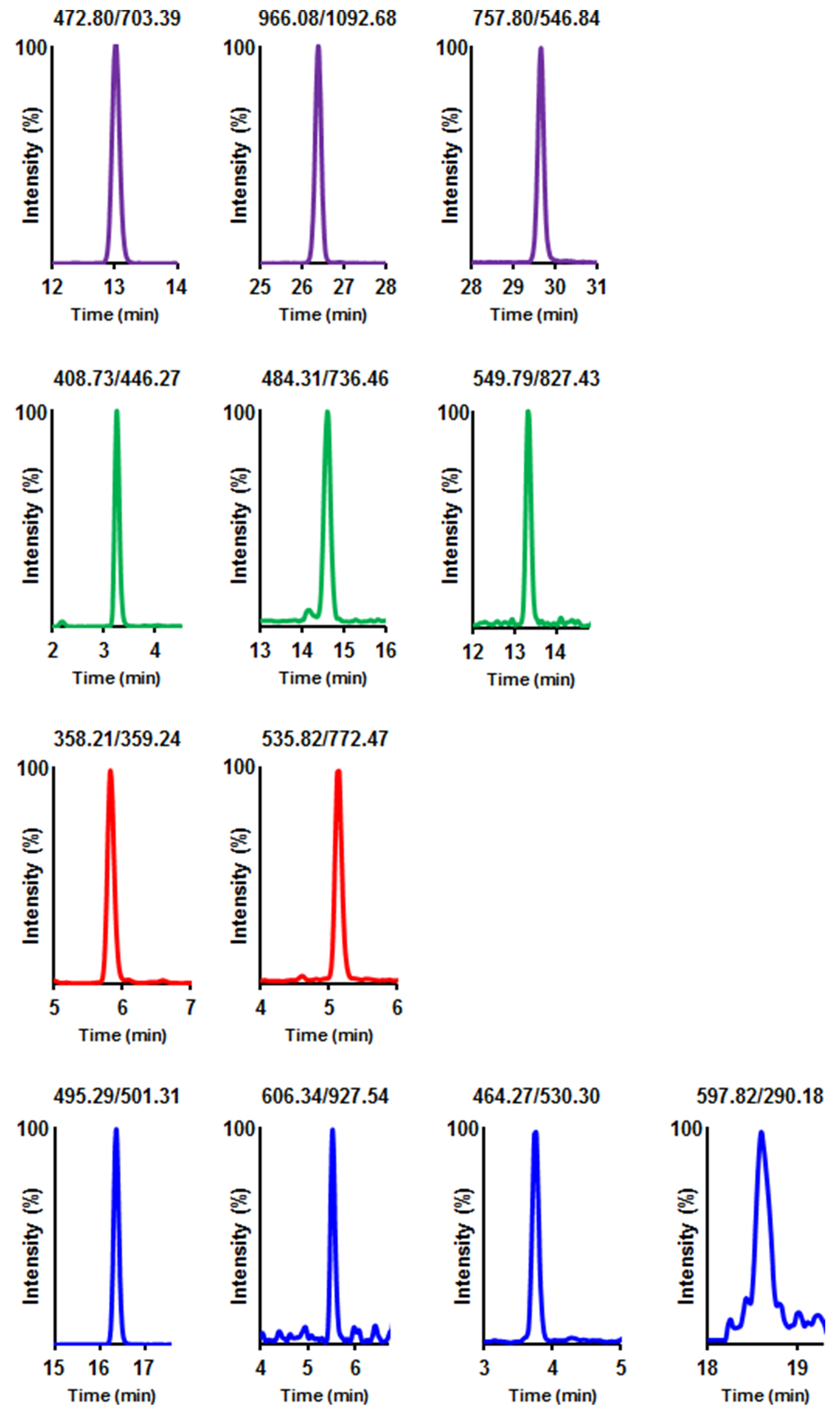

Supplement: Supplementary file 3 — 10.1186/s12014-015-9098-1 Chromatograms of transitions used for quantification. Transitions for H2A (purple), H2B (green), H3 (red), and H4 (blue) with non-modified peptides used for normalization in the first column. [file 12014_2015_9098_MOESM3_ESM.tif]
